# Supplementary material for: Enhancement of Stability in n-Channel OFETs by Modulating Polymeric Dielectric
Source: Polymers (Basel). 2023 May 23;15(11):2421. doi: 10.3390/polym15112421 (PMC10255236; doi:10.3390/polym15112421)
Supplement: Supplementary file 1 [file polymers-15-02421-s001.zip › polymers-2360931-supplementary.pdf]

Enhancement of stability in *n*-channel OFETs by modulating polymeric dielectric

Po-Hsiang Fang, [1] **Peng-Lin Kuo,** [1] **Yu-Wu Wang,** [2] **Horng-Long Cheng,** [1] **and Wei-Yang Chou,** [1,*]

1 Department of Photonics, National Cheng Kung University, Tainan 70101, Taiwan

2 Graduate Institute of Photonics, National Changhua University of Education, Changhua 50007, Taiwan

*Correspondence: weiyang@ncku.edu.tw

Table S1. Performance criteria of OFETs with different dielectrics (averaging by 10 devices).

Dielectric of OFETs	V_{th} (V)	I_{On}/I_{Off} Ratio	*S.S.* (V/dec)	Mobility (cm^2/Vs)
PI (6.4 wt%) /AlO_x	-0.83 ± 0.41	(3.8 ± 3.4) × 10^3	0.76 ± 0.45	(2.5 ± 1.7) × 10^{-3}
PI (4.7 wt%) /AlO_x	-0.26 ± 0.16	(1.5 ± 1.2) × 10^4	0.74 ± 0.31	(2.3 ± 1.4) × 10^{-3}
PI (3.2 wt%) /AlO_x	0.16 ± 0.12	(2.5 ± 2.4) × 10^4	0.34 ± 0.15	(1.0 ± 0.7) × 10^{-2}
PI (1.6 wt%) /AlO_x	0.13 ± 0.09	(1.3 ± 1.0) × 10^4	0.40 ± 0.10	(1.6 ± 1.1) × 10^{-2}
AlO_x	0.15 ± 0.04	(1.9 ± 1.7) × 10^2	1.35 ± 0.95	(1.5 ± 0.6) × 10^{-4}

Table S2. Comparison of mobilities between different references and this work.

Semiconductor	Mobility (cm^2/Vs)	Ref.
[5] phenacene	1.5 × 10^{-3}	35
P(NDI2OD-T_2)	3.4 × 10^{-3}	36
PTCDI-C_{13}	2.3 × 10^{-2}	37
$C_3F_7CH_2$-PTCDI-$(CN)_2$	4.0 × 10^{-2}	38
PTCDI-C_{13}	1.4 × 10^{-2}	39
PTCDI-C_{13}	1.6 × 10^{-2}	40
PTCDI-C_{13}	2.7 × 10^{-3}	41
4Cl-PDI-3EG	8.0 × 10^{-4}	42
PTCDI-C_{13}	1.5 × 10^{-2}	This work

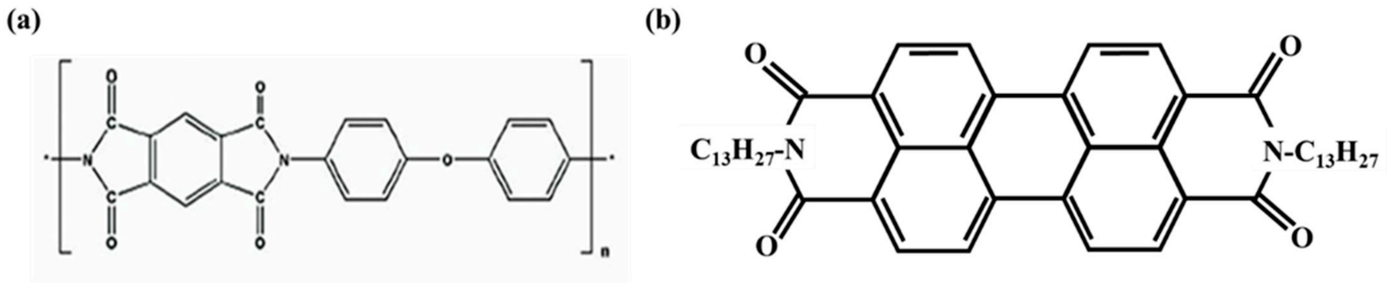

Figure S1. The chemical structures of (a) polymer dielectric material PI, and (b) organic semiconductor material PTCDI-C_{13}.

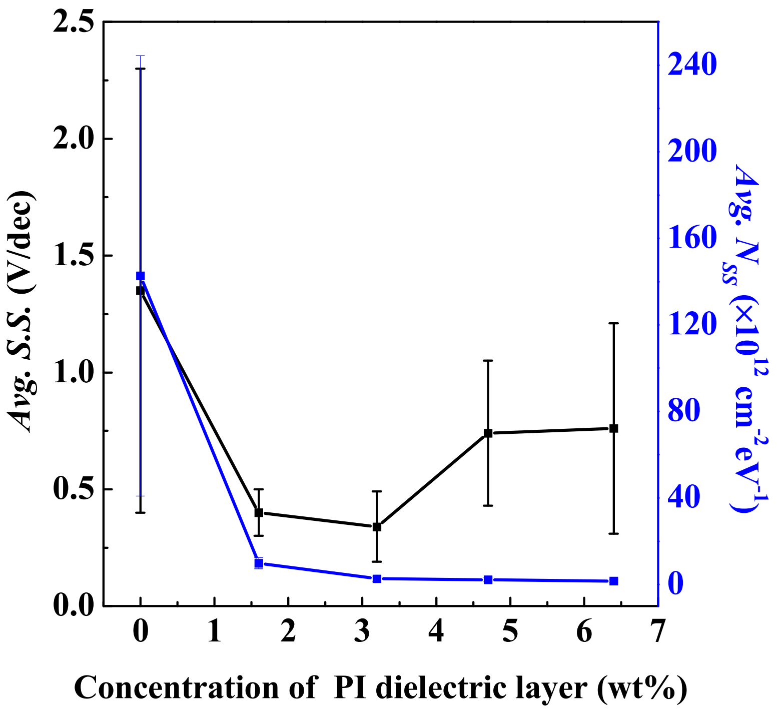

Figure S2. The relationship between the *S.S.* and N_{SS} of the OFETs versus the different solid contents of the PI layer, in which 10 devices are used for averaging.

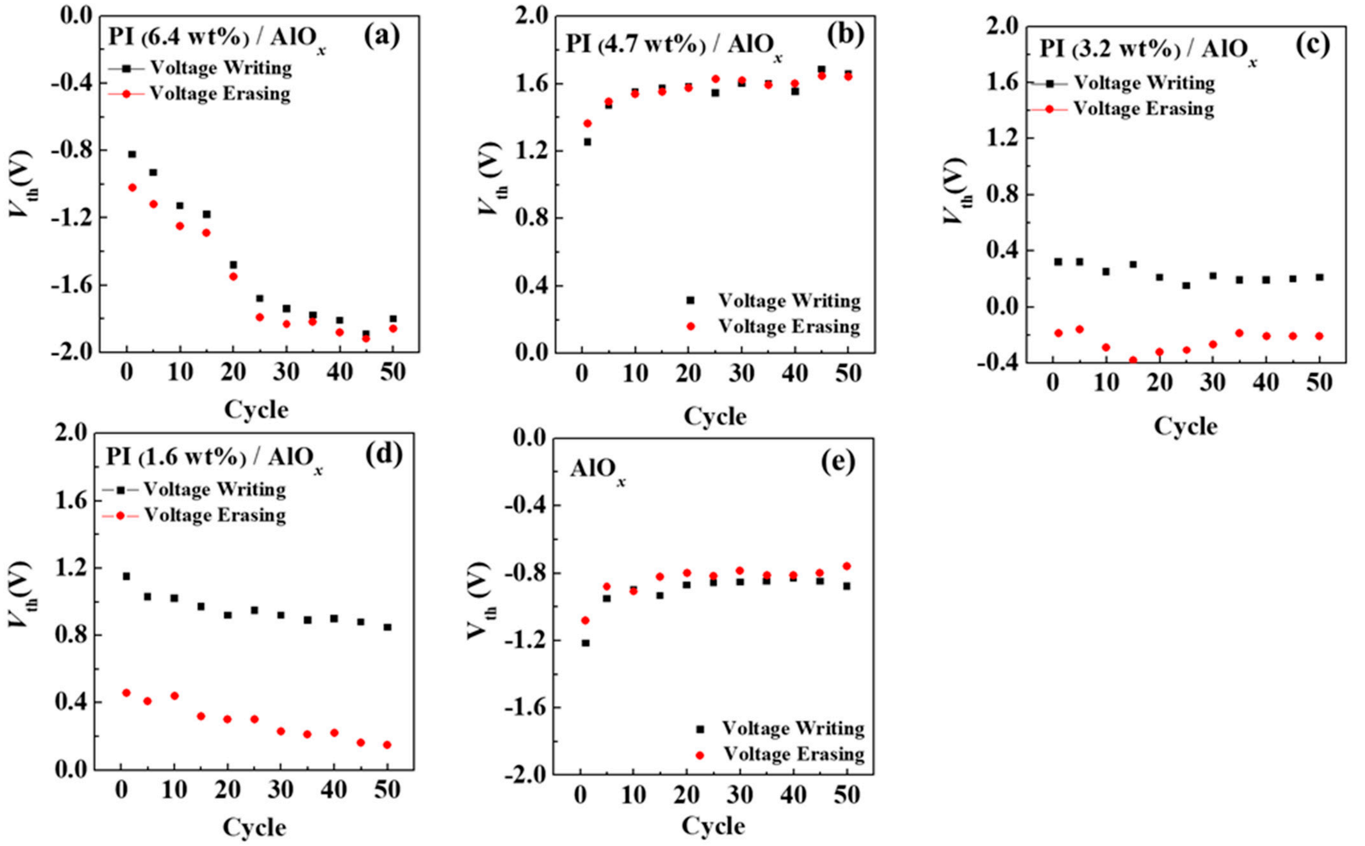

Figure S3. The V_{th} variation chart of writing-erasing for organic memory devices fabricated by using PI films with different solid contents (a) 6.4 wt%, (b) 4.7 wt%, (c) 3.2 wt%, (d) 1.6 wt%, and (e) native AlO$_x$.

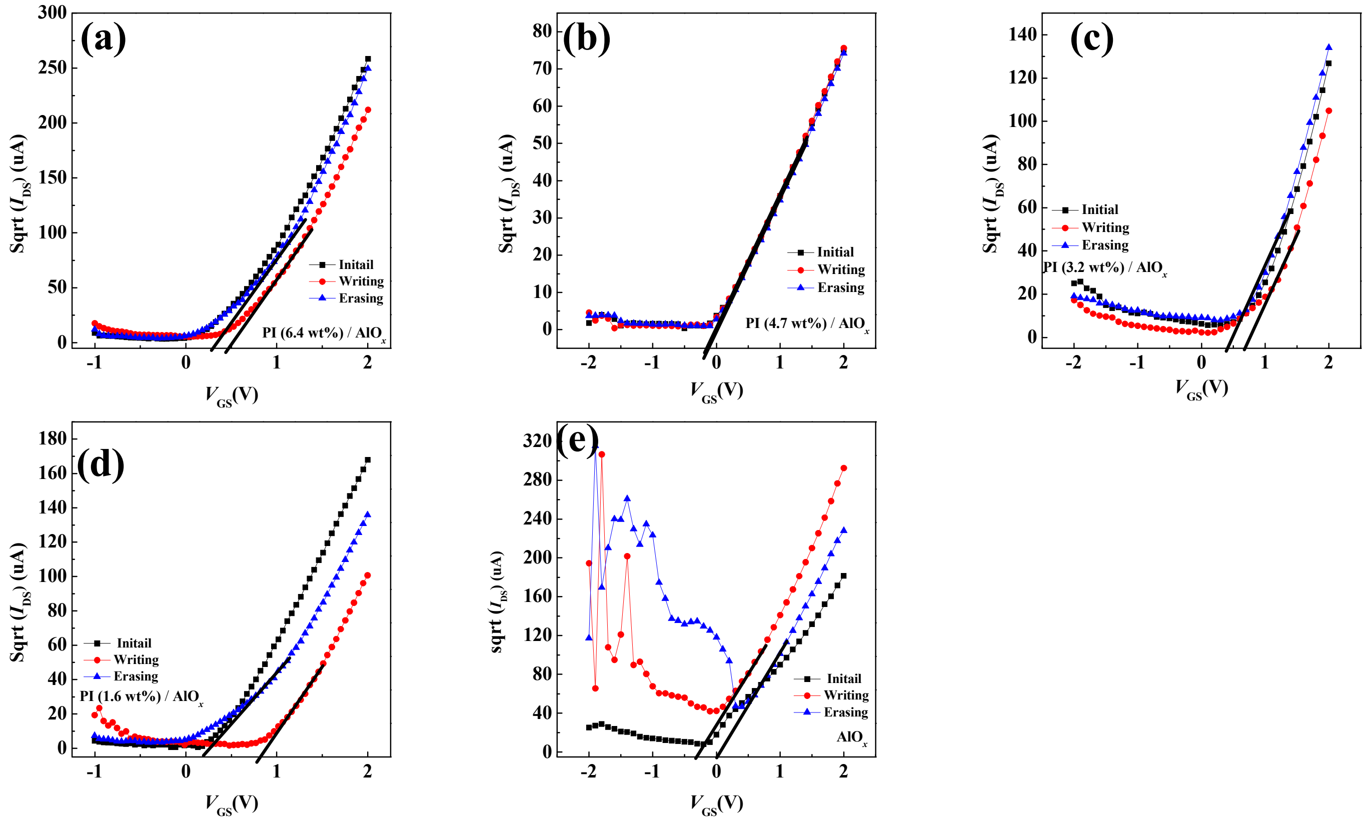

Figure S4. The memory characteristics chart of writing-erasing behaviors for organic memory devices fabricated by using PI films with different solid contents (a) 6.4 wt%, (b) 4.7 wt%, (c) 3.2 wt%, (d) 1.6 wt%,

and (e) native AlO_x.
